# Genome Wide Association Study to Identify the Genetic Base of Smallholder Farmer Preferences of Durum Wheat Traits

**DOI:** 10.3389/fpls.2017.01230

**Published:** 2017-07-17

**Authors:** Yosef G. Kidane, Chiara Mancini, Dejene K. Mengistu, Elisabetta Frascaroli, Carlo Fadda, Mario Enrico Pè, Matteo Dell'Acqua

**Affiliations:** ^1^Institute of Life Sciences, Scuola Superiore Sant'Anna Pisa, Italy; ^2^Sirinka Agricultural Research Center Woldia, Ethiopia; ^3^Bioversity International Addis Ababa, Ethiopia; ^4^Department of Dryland Crop and Horticultural Sciences, Mekelle University Mekelle, Ethiopia; ^5^Department of Agricultural Sciences, University of Bologna Bologna, Italy

**Keywords:** GWAS, folk wisdom, traditional knowledge, small farming, smallholder farmers, QTL mapping, landraces, *Triticum*

## Abstract

Smallholder agriculture involves millions of farmers worldwide. A methodical utilization of their traditional knowledge in modern breeding efforts may help the production of locally adapted varieties better addressing their needs. In this study, a combination of participatory approaches, genomics, and quantitative genetics is used to trace the genetic basis of smallholder farmer preferences of durum wheat traits. Two smallholder communities evaluated 400 Ethiopian wheat varieties, mostly landraces, for traits of local interest in two locations in the Ethiopian highlands. For each wheat variety, farmers provided quantitative evaluations of their preference for flowering time, spike morphology, tillering capacity, and overall quality. Ten agronomic and phenology traits were simultaneously measured on the same varieties, providing the means to compare them with farmer traits. The analysis of farmer traits showed that they were partially influenced by gender and location but were repeatable and heritable, in some cases more than metric traits. The durum wheat varieties were genotyped for more than 80,000 SNP markers, and the resulting data was used in a genome wide association (GWA) study providing the molecular dissection of smallholder farmers' choice criteria. We found 124 putative quantitative trait loci (QTL) controlling farmer traits and 30 putative QTL controlling metric traits. Twenty of such QTL were jointly identified by farmer and metric traits. QTL derived from farmer traits were in some cases dependent on gender and location, but were consistent throughout. The results of the GWA study show that smallholder farmers' traditional knowledge can yield QTL eluding metric measurements of phenotypes. We discuss the potential of including farmer evaluations based on traditional knowledge in crop breeding, arguing for the utilization of this untapped resource to develop better adapted genetic materials for local agriculture.

## Introduction

Through time, plant breeding has adapted crops to societal needs. Since the invention of agriculture more than 10,000 years ago (Diamond, 2002), a pressing selection for favorable traits have been exerted on the allelic pools of major crops (Purugganan and Fuller, 2009). Those plant features addressing the needs of agriculture were identified and promoted, shaping nowadays crops. With the progress of agriculture, the methods available to produce better plants radically changed. The quantitative evaluation of crop phenotypes moved from an unconscious process based on visual assessment of plant traits to the most recent field (Fahlgren et al., 2015) and greenhouse (Li et al., 2014) sensing platforms. Concurrently, genotyping and genome sequencing technologies provided the means to dramatically increase inter-generation genetic gain through quantitative trait loci (QTL) mapping and cloning (Fu et al., 2009), marker assisted selection (Lande and Thompson, 1990), and genomic selection (Goddard and Hayes, 2007). The requirement of an increasingly complex technology to produce better crops sided the shift from subsistence farming to industrialized agriculture and centralized breeding (Evenson and Gollin, 2003; Borlaug, 2007). Much of the world, however, still lays outside the benefits of highly productive and profitable modern varieties (MV) introduced since the green revolution (Diao et al., 2008), either because of poor seed circulation or because farmers still prefer to grow their traditional varieties over MVs poorly adapted to local agriculture (Jarvis et al., 2011).

As many as 900 million of world's poor people live and work as smallholder farmers in rural areas exposed to harsh and low-input farming conditions and to dramatic climate change effects (International Fund for Agricultural Development, 2001; Morton, 2007). A vivid example of the smallholder farming system in Sub-Saharan Africa is Ethiopia, where 8 out of 10 persons in the population of the country now approaching 100 million people are involved in farming (FAO Statistics Division, 2015), and more than 80% of the farmers are smallholders (Salami et al., 2010). Ethiopian farming communities typically conduct mixed farming, keeping animals and growing cereals, pulses and oil crops in small land parcels. Despite the productivity of the Ethiopian farming system is highly exposed to shifts in climate and weather (Mann and Warner, 2017), Ethiopia is the biggest wheat producer in Sub-Saharan Africa (FAO Statistics Division, 2015). Primary wheat production constrains in Ethiopia are poor access to inputs, fertilizers, and quality seeds, that are employed on a fraction of the total area cultivated (Bergh et al., 2012). Most of the Ethiopian farming communities inhabit fragile landscapes with poor soils, erratic rainfalls, and modest connections to markets. Seeds are exchanged through an informal seed system that favors the spread and maintenance of locally adapted landraces. Although new varieties are released every year by national and international breeding efforts, the deployment of MVs in smallholder fields is hampered by poor distribution and poor farmers' uptake, especially due to MVs failure to address farmers' needs and to their poor adaptation to marginal growing conditions.

In subsistence systems, farmers are still entrusted with the selection of the varieties capable of sustaining the household in the following season. Their choice of varieties relies on traditional knowledge and past field experiences, and determines food security at the household level. Smallholder farmers must be efficient in determining whether crop varieties suit their needs and the agroecology they inhabit. For these reasons, smallholder farmers are knowledgeable of traits concerning environmental adaptability such as resistance to drought, frost, pests, and diseases (Asrat et al., 2010), as well as of agro-morphological traits such as number of spikes and seeds produced (Elmyhun and Mekonen, 2016). The varietal evaluation given by farmers may not overlap to that of breeders (Burman et al., in press). In some cases, preferred traits can differ among locations and gender groups: women are often more concerned with filling the food security gap and may have a preference for cooking related traits, whereas men are more concerned with field problems and market demand and tend to prefer traits more related with yield stability and productivity (Assefa et al., 2014; Kolech et al., 2015).

Traditional knowledge has already benefited several fields of quantitative sciences. The poison on Amazonians' arrows has become antidepressants (Feldman, 2009), and willow bark extracts have been made into anti-inflammatories (Mahdi et al., 2006). Hundreds of plant-derived medical compounds have the same or related purposes as their ethnomedical history suggests (Fabricant and Farnsworth, 2001). Here, we advocate that smallholder farmers' traditional knowledge could be similarly harnessed for the benefit of local and global wheat breeding, through the identification of loci contributing to wheat desirability by the farmers themselves. Our results show that farmer evaluations are measurable and repeatable, and can be used in a genome-wide association (GWA) study to close the loop between farmers' traditional knowledge and modern breeding. Our study focuses on two separate smallholder farming communities in Ethiopia. Farmers expressed their preferences on durum wheat genotypes based on previously defined traits, and their evaluation was used to determine the genetic basis of their preferences. The aim of this study is not to individually discuss the QTL identified by GWA on either farmer traits and metric traits, but rather to report the congruity (or lack thereof) among the two and to demonstrate the feasibility of incorporating farmers' traditional knowledge in molecular breeding methods.

## Materials and methods

### Experimental sites

The study was conducted during the 2012 wheat growing season in two locations in the Ethiopian highlands. The first location was in the Geregera area, in the village of Workaye, Meket district (Amhara region, 11°40′N/38°52′E, WGS84; hereafter identified as Geregera). The second location was in the Hagreselam district, in the village of Melfa (Tigray region, 13°39′N/39°10′E, WGS84; hereafter Hagreselam). The two locations are representative of Ethiopian wheat growing areas at high (2,867 m.a.s.l.) and medium (2,572 m.a.s.l.) altitudes, respectively. On average, households in Geregera and surrounding areas are 1.43 ha in size and are composed by 5.5 members. In Hagreselam, households have the same membership (5.5 people on average) but cover only 0.6 ha per household. Livestock is usually part of the farm in both locations: tropical livestock units for smallholder farmers in Geregera are 2.6, while in Hagreselam are 2.1.

### Farmer selection and focus group discussions

In each location, 30 smallholder farmers growing wheat were selected on a voluntary basis, but keeping a 50:50 gender representation. Both man and women involved in the study were themselves durum wheat growers, and belonged to different households so to avoid family bias. Among volunteers, the farmer panel was assembled to avoid bias in gender, age, and wealth. Farmers' age ranged from 22 to 46 years in Geregera and from 26 to 70 years in Hagreselam. Farmer groups were divided by gender to account for potential differences in wheat evaluation, and were involved in focus group discussions (FGD) at each location. At the FGDs onset, we collected demographic information on the participants. All participants provided written informed consent to participate in the research. Ethics approval was not required as per institutional and national guidelines. Researchers speaking the local language (Amharic in Geregera and Tigrinya in Hagreselam) moderated FGDs. Farmers were asked to list the traits they used to evaluate wheat varieties, hereafter termed farmer traits (FT), and these were ranked by importance. Among the most important and recurring traits indicated by farmers, we chose traits that could also be evaluated in the experimental fields running at the time of the evaluation. Processing traits such as cooking and baking quality could not be assessed in the field, thus were excluded from the downstream evaluation. Three FTs were selected: (i) earliness, as the maturation stage at the time of the field evaluation, (ii) tillering capacity, as the capacity to produce secondary stems and spikes, and (iii) spike morphology, as the overall appearance of the spike. Additionally, the overall appreciation, i.e., the overall evaluation of the desirability of a specific genotype, was added to FTs as synthetic criterion of farmers' preferences.

### Plant material and field design

The genetic material here analyzed is a diversity panel comprising 400 Ethiopian wheat accessions conserved *ex situ* at the Ethiopian Biodiversity Institute (EBI; http://www.ebi.gov.et/). The farmer communities did not have prior access to such genotypes, and were oblivious of the material to be tested. All the accessions included in the diversity panel had at least partial passport data. Twenty-eight accessions among them were so-called “improved varieties,” i.e., MVs released for cultivation in Ethiopia. The remaining were Ethiopian wheat landraces. The year prior to the field experiment, each accession was grown and inspected for variability. In order to exclude heterogeneity within landraces, a reference spike for each accession was selected as the standard genotype, and was used to amplify the seeds required for the subsequent field experiments and for the DNA extraction. The diversity panel was sown in Geregera and Hagreselam following a replicated 20 × 20 partial lattice design at a seed rate of 100 kg ha^−1^. Both sites are test fields commonly used for cereals relevant for Amhara and Tigray regions of Ethiopia, respectively. Crops frequently cultivated in both locations include barley, chickpea, faba bean, field pea, lentil, and teff. Rainfall in Geregera is annually 1,300 mm, yet typically erratic, and soil is mainly lithosol. In Hagreselam the annual rainfall is 680 mm, and soils are mainly clay loam. The experimental fields were designed to provide standardized conditions to evaluate genotypes' performance. Planting date was July the 5th, 2012 in Geregera, July the 7th, 2012 in Hagreselam. In both locations land was prepared by oxen plowing, and planting was performed manually by drilling. The plot size was 2.5 × 0.8 m, each plot having four rows of plants. The middle two rows were used for data collection. Spacing between rows and replications was 0.5 and 1.5 m, respectively. Field management, in rain-fed conditions, was uniform at the two locations. Doses of 46 kg P_2_O_5_ ha^−1^ fertilizer in the form of DAP and 41 Kg N ha^−1^ in the form of Urea and DAP were applied during sowing. Additional 23 Kg N ha^−1^ were applied in the form of Urea at the beginning of tillering in both sites. Weeds were controlled manually. Field management in the experimental sites differed from that traditionally used by smallholder farmers especially in regards of fertilizers and intensity of manual weeding (lower in farmer fields), and seed rate (higher in farmer fields). For further details on material selection and field design, see Mengistu et al. (2016).

### Genotyping

DNA extraction was conducted in Ethiopia, at the Mekelle University Molecular and Biotechnology Laboratory (Mekelle, Tigray). Five seedlings were germinated and pooled for each accession. Genomic DNA was extracted from green tissues with the GenElute Plant Genomic DNA Miniprep Kit (Sigma-Aldrich, St Louis, MO) according to the manufacturer's directions. Quality was checked on Nanodrop 2000 (Thermo Fisher Scientific Inc., Waltham, MA) and by electrophoresis on 1% agarose gel. Genotyping was performed on the Infinium 90K wheat chip (Wang S. et al., 2014) at TraitGenetics GmbH (Gatersleben, Germany). The molecular markers thus produced were filtered for minor allele frequencies above 5% and failure rate below 20% with custom scripts in R (R Development Core Team, 2017). Detailed molecular diversity analyses conducted on the diversity panel are reported in Mengistu et al. (2016).

### Metric traits collection

Technicians measured 10 metric traits (MT) in each location. Days to 50% booting (DB), days to 50% flowering (DF), and days to 75% maturity (DM) were measured for whole plots. Number of effective tillers per plant (NET), plant height (PH, in cm), spike length (SPL, in cm), the number of seeds per spike (SPS) were measured on three randomly selected plants per plot. Grain yield (GY; grams of grain produced per plot, converted in t ha^−1^), above ground biomass or biological yield (BY; dry weight of the above ground harvested biomass grams per plot, in t ha^−1^) and thousand grain weight (TGW; weight of 1,000 kernels, in grams) were measured on full plots. For further details on metric phenotypes collection, see Mengistu et al. (2016).

### Farmer traits collection

The procedure for FTs collection was identical at the two locations. At early seed maturation, when flowering time differences were still visible, the 30 farmers were organized into smaller groups of five members of the same gender. A rapporteur, a local technician with agronomic training, accompanied each group. Groups were led into the field from random access points and were taken to one plot at a time following a different path. The average maturing stage in Geregera on Zadoks scale was 75 (medium milk), in Hagreselam was 85 (soft dough). For each plot, the group had to score the four FTs from 1 to 5, where 1 was poor and 5 excellent. The scoring was conceived as the answer to the following question: “what is your evaluation of [the FT] of this plot?” Plots were labeled anonymously. In order to prevent farmers from influencing each other, each farmer was given five seeds (each representing a scoring unit) and asked to hold them in her/his closed hand. At each plot, and for each FT, the farmer had to pick a number of seeds equivalent to their score of the trait without letting others see her/his choice. Immediately, farmers were instructed to open their hands and show their score, which was individually noted by the rapporteur.

### Statistical analyses

An ANOVA was conducted on phenotypic data collected in each location. A mixed model was used, including genotype, replication, incomplete block within replication effects, and the residual error. The ANOVA combined over locations was performed including also location, and genotype by location interaction as fixed factors. For the calculation of genotypes adjusted means, best linear unbiased estimates (BLUEs) were computed by considering all effects as random except the expected mean and the genotypic effect. Computations were performed using PROC MIXED, expected mean square method (Type3), in SAS (SAS Institute, Cary, NC). Heritability (*h*^2^) for each trait in each location was calculated on a genotype-mean basis across the two replications (r), as $\sigma_{\text{g}}^{2}$/($\sigma_{\text{g}}^{2}$ + $\sigma_{\text{e}}^{2}$/r), where $\sigma_{\text{g}}^{2}$, and $\sigma_{\text{e}}^{2}$ are the genotype and the residual error variances, respectively. The phenotypic correlation (*r*_p_) was calculated among all FTs and MTs traits collected in Hagreselam and Geregera and for each trait between locations. Pearson's correlations were calculated with SAS (SAS Institute, Cary, NC). Correlation plots were produced with the R package R/corrplot (Wei, 2013). A principal component analysis (PCA) was performed on location-specific and across-location MTs to extract the most variable axes of phenotypic variation as principal components (MT-PCs). The MT-PCs explaining the highest variance were correlated back to MTs. Phenotypic MT-PCs were retained and sided to metric phenotypes for further analyses. All plots were produced with R custom scripts available upon request.

Upon the molecular analysis reported in Mengistu et al. (2016), 312 samples were classified as durum wheat. These samples, further filtered for missing data, were the sole used for the GWA study. The filtered set of polymorphic, high-quality molecular markers scored on these samples was input in the R package Genome Association and Prediction Integrated Tool (GAPIT) (Lipka et al., 2012). Only polymorphic markers with a genetic position on the durum wheat genetic map (MacCaferri et al., 2015) were retained. The GWA scan was run on FTs and MTs, and for the most important MT-PCs. R/GAPIT was run under the SUPER method (Wang Q. et al., 2014). Population structure was corrected using a kinship matrix calculated with the VanRaden method (VanRaden, 2008) and principal components deriving from molecular marker data (SNP-PC) as covariates. The GWA scan was iteratively run with 1–10 SNP-PCs as covariates, and quantile-quantile plots were visually evaluated to choose the best fit of the model, that is the sole reported. Multiple test correction was performed according to the Bonferroni method on a nominal test *p*-value of 0.05. Tests surpassing this significant threshold denote significant marker trait associations (MTA). A regression model was fitted for each MTA between marker allele scores (arbitrarily set to −1 for homozygous for the highest frequency allele, 0 for heterozygous, and 1 for homozygous for the lowest frequency allele) and the phenotypic values to estimate the MTA effect and its *R*^2^. Significant MTAs were grouped on the basis of LD decay information calculated on the same set of markers on the same genotypes panel (Mengistu et al., 2016). When multiple MTAs were falling within chromosome-specific LD halving distance from each other, they were assigned to the same putative QTL. Custom R script were used to analyze the overlap and distribution of FT and MT putative QTL. GWA plots in the main text were created with custom R scripts available upon request. Manhattan plots where produced with R/qqman (Turner, 2014), quantile-quantile plots where produced with modified R/GAPIT functions. Correlation plots were produced with R/corrplot (Wei, 2013).

## Results

The 400 wheat genotypes were evaluated for four FTs and 10 MTs. In each location, genotype variance was significant for most of the traits, except for NET (Table S1). Since in the combined analysis location by genotype interactions were significant for the large majority of FTs and for several MTs (Table 1), the locations were kept separated in the subsequent analyses. Still, measures of the same trait collected in the two locations were always significantly correlated (Table 1), with the highest values of *r*_P_ for Earliness and Spike FTs, and for DB and PH among the MTs. Overall evaluation, a composite measure, reached an *r*_P_ of 0.585 for men. For comparison, the *r*_P_ for GY in the two locations was just 0.424 (Table 1). Correlations among FTs and MTs within locations (Table S2) showed that the overall appreciation, arguably the most composite trait provided by farmers, was highly correlated with spike morphology FT, and with plant height, biomass, grain yield and yield components such as thousand grain weight and number of seeds per spike (Figure S1). In each location, the proportion of phenotypic variation due to genetic variation, estimated as heritability (*h*^2^), was for FTs comparable to those of MTs. Among FTs, *h*^2^ was lower for tillering capacity and higher for earliness. Earliness is expectedly the FT with the highest *h*^2^ (Table S1): this trait is easy to measure in open fields, and highly correlated with the metric measurement of DB, DF, and DM (Figure S1).

**Table 1:** Significance of variance for genotype (G), location (L), and location by genotype (LxG) interactions for farmer traits (FT) and metric traits (MT) combined across locations. Error is given for L. For each trait are given the mean (Grand Mean), the minimum value (Min), the maximum value (Max), and the phenotypic correlation among locations (*r*_p_).

**Farmer Traits (MT)**		**Overall**		**Earliness**		**Spike morphology**		**Tillering capacity**			
	**d.f**.	**Women**	**Men**	**Women**	**Men**	**Women**	**Men**	**Women**	**Men**		
**SOURCE OF VARIATION**											
Location (L)	1	198.599^*^	87.119	334.743	117.840	297.778^*^	2.083	100.994^*^	4.794		
Error^a^	2	5.569	10.610	40.083	7.933	3.487	10.462	4.866	14.963		
Genotype (G)	399	0.437^**^	0.863^**^	1.509^**^	2.018^**^	0.498^**^	0.788^**^	0.344^**^	0.342^**^		
L × G	399	0.213^**^	0.231^**^	0.396^**^	0.359^**^	0.162^**^	0.153	0.153	0.156^*^		
Residual	715-722	0.136	0.148	0.209	0.240	0.132	0.134	0.152	0.133		
Grand Mean		3.13	2.78	3.35	3.03	3.31	2.95	3.06	2.71		
Min		1.98	1.68	1.25	0.99	2.19	1.79	1.90	1.68		
Max		4.03	4.40	4.51	4.43	4.26	4.19	3.86	3.60		
*r*_P_		0.434	0.585	0.600	0.699	0.550	0.681	0.406	0.402		
**Metric Traits (MT)**	**d.f**.	**DB**	**DF**	**DM**	**GY**	**BY**	**NET**	**PH**	**SPL**	**SPS**	**TGW**
**SOURCE OF VARIATION**											
Location (L)	1	25721^*^	47012^*^	173373^**^	284.14^*^	88.03	8189.14^**^	11085	228.799^*^	8466.73^**^	2482.72^*^
Error^a^	2	451	569	73	3.260	97.58	2.80	1677	9.784	77.5	63.7
Genotype (G)	399	81.34^**^	73.34^**^	94.5^**^	0.68^**^	5.728^**^	2.19^**^	338.12^**^	2.458^**^	74.99^**^	52.57^**^
L × G	399	13.15^**^	15.18^**^	29.91^**^	0.29	2.696	1.65	56.88	0.642^*^	26.53	11.44
Residual	715-722	8.28	8.97	22.38	0.30	2.875	1.74	66.54	0.536	31.15	11.50
Grand Mean		71.24	83.31	128.97	2.12	6.72	4.81	97.40	7.47	31.48	35.25
Min		56.32	74.10	111.65	0.69	3.27	2.70	62.16	4.98	18.51	25.06
Max		88.59	100.33	144.35	3.43	10.52	7.24	119.76	9.68	50.97	45.04
*r*_P_		0.737	0.672	0.567	0.424	0.362	0.213	0.708	0.612	0.491	0.675

a Error for testing Location. Significance of the F-test:

* *p < 0.05*,

** *p < 0.01; d.f. range of residuals according to the number of missing values per phenotype. DB, days to booting; DF, days to flowering; DM, days to maturity; GY, grain yield; BY, biological yield; NET, number of effective tillers; PH, plant height; SPL, spike length; SPS, seeds per spike; TGW, thousand grain weight*.

**Figure 1:**
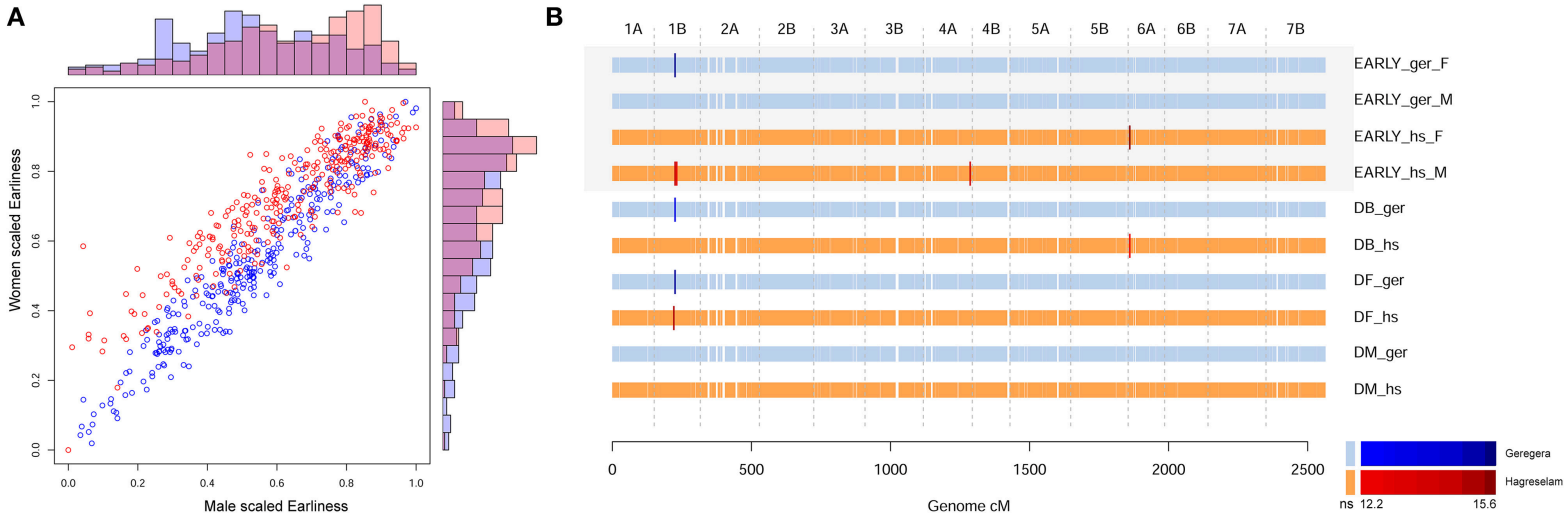
GWA scan of earliness evaluated by farmers. **(A)** Comparison of earliness scores among genders and locations, scaled. The scatter plot reports the covariance of men (x-axis) and women (y-axis) scores in each location, blue for Geregera and red for Hagreselam. The outer bar plots compare the earliness score distribution within genders and among locations. The top plot compares men from Geregera (blue) and Hagreselam (red). The side plot similarly depicts women scores. **(B)** GWA scan on earliness (EARLY), days to booting (DB), days to flowering (DF), and days to maturity (DM) in both locations. Each horizontal line represents a GWA scan, next to the corresponding trait name. Men and women's scores are kept separate, denoted by “M” and “F,” respectively. Ticks represent markers ordered by genetic position as reported on the x-axis. Brighter, bigger ticks represent significant associations with a color code reported in the legend below. GWA for Geregera (ger) traits are in shades of blue, GWA for Hagreselam traits (hs) are in shades of red.

**Figure 2:**
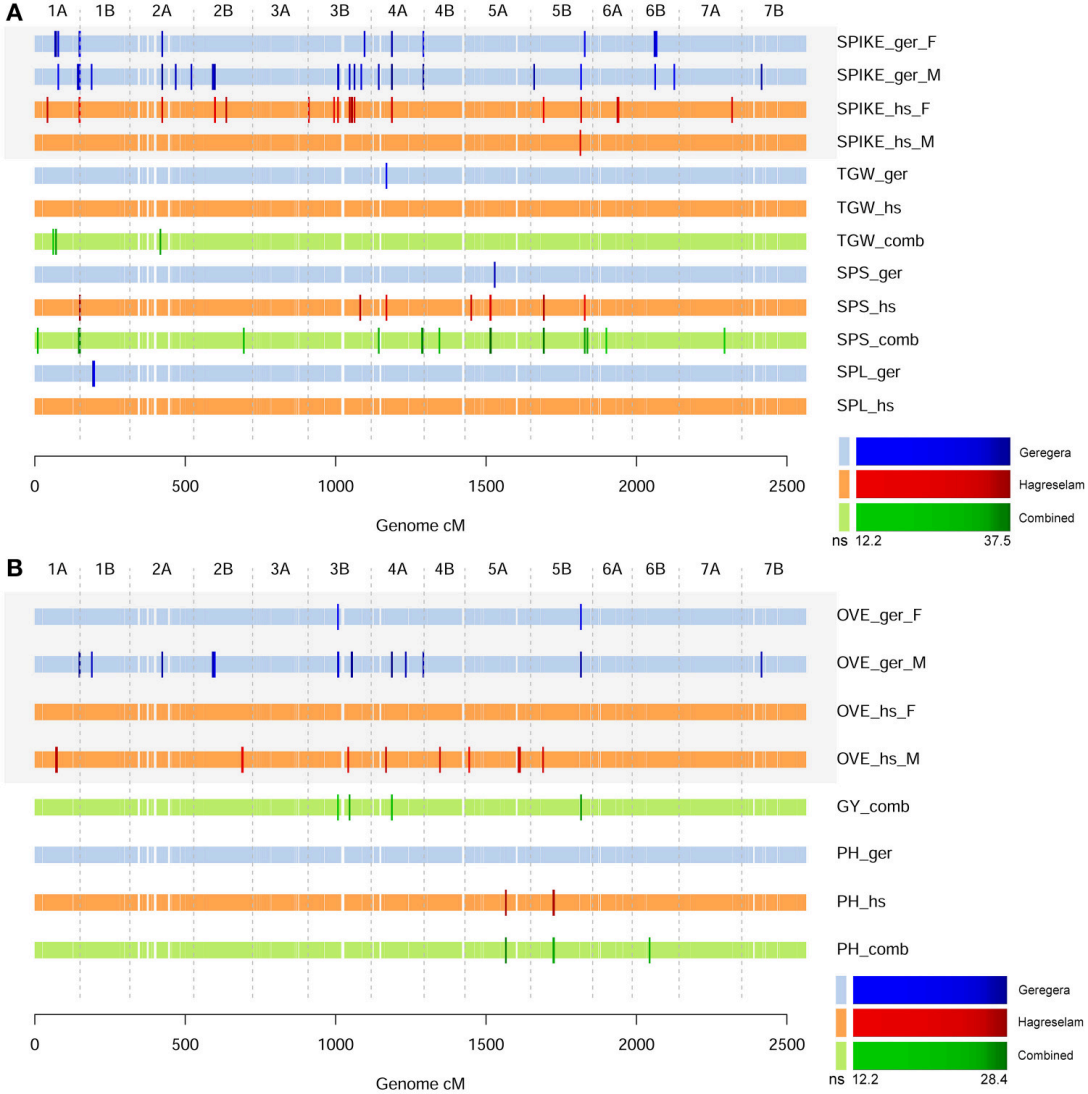
GWA scans for spike and overall farmer traits. **(A)** The GWA scan for spike morphology (SPIKE), the number of seeds per spike (SPS), spike length (SPL), and thousand grain weight (TGW). GWA for Geregera traits (ger) are shown in shades of blue. GWA scans on metric values derived from the combination of traits collected in each location are shown in shades of green. GWA for Hagreselam traits (hs) are shown in shades of red. Brighter, bigger ticks represent significant associations with a color code reported in the legend below. **(B)** The GWA scan for overall evaluation (OVE), grain yield (GY), and plant height (PH), represented as in **(A)**.

The evaluation of earliness was slightly different among locations and among genders within the same location, with the highest variability in Hagreselam (Figure 1A). The marker-trait associations (MTA) identified for DB and DF in either or both locations combined (Mengistu et al., 2016) were jointly identified by earliness FT (Figure 1B; Table S3). An MTA on chromosome (Chr) 1B was identified by earliness FT and related MTs in both locations. Further MTAs specific to these traits collected in the Hagreselam location emerged on Chr 4B and 6A. Several suggestive associations, although not surpassing the stringent significance threshold, overlap these MTAs (Figure S2). The GWA on spike morphology (Figure S3) revealed several MTAs jointly identified by the two communities and overlapping MTAs derived from spike-related MTs (Figure 2A; Table S4). Although, quantile-quantile plots for spike traits showed some inflation (Figure S4), the strongest MTAs were consistent across traits and locations. These included two MTAs on Chr 1A, and several MTAs on Chr 3B, 4A, and 5B (Figure S4). The GWA scan on overall evaluation provided fewer MTAs (Figure 2B; Table S5), possibly because of more diverse scores across genders and communities (Figure S5). Four MTAs for combined measures of GY were identified by farmers in Geregera, and only one in Hagreselam. The most consistent MTAs in Geregera emerged on Chr 3B and 5B (Figure S6), the latter in common with spike FT (Figure 2A). A suggestive peak overlapping the latter was also identified by women in Hagreselam yet did not surpass the significance threshold (Figure S6). Typically, women provided fewer MTAs than men. This is matched by the consistently lower *r*_p_ and *h*^2^ shown by women-scored FT (Table 1, Table S1) and by more skewed and varied evaluations (Figure S7). When detected, however, the position of women's MTAs matched that of men, as in the case of Chr 3B and 5B. The tillering capacity FT (Figure S8) provided just one MTA detected on Chr 2B by women in Hagreselam (Figure S9). The measured NET did not report MTAs, although a suggestive signal overlapping the MTA identified by tillering FT was present in Hagreselam (Figure S10, Table S6). This is likely due to the low *h*^2^ of the trait (Table S1). The MTAs resulting from the GWA scan of FTs and MTs were joined according to chromosome-specific LD decay measures, yielding a total of 134 putative QTL (Table S7). Of these, 124 were identified for FTs and 30 for MTs. Twenty of such putative QTL (14.9%) were jointly identified for both FTs and MTs. The traits providing more putative QTL where spike and overall quality assessed by farmers (105 and 83, respectively). In most of the cases, MTAs were identified by multiple traits. This is the case of spike and overall FTs, which in 71 instances identified the same putative QTL (Table S8). Most of the putative QTL could be traced to just one MTA, and these in turn were mainly derived from FTs. Two thirds of the MTs putative QTL were identified for FTs as well (Figure S11). Putative QTL from FTs explained as much as 19% of the phenotypic variation in case of spike morphology, 15% in case of overall evaluation. Number of seeds per spike provided the MTA with the highest *R*^2^, 22% (Table S9).

**Figure 3:**
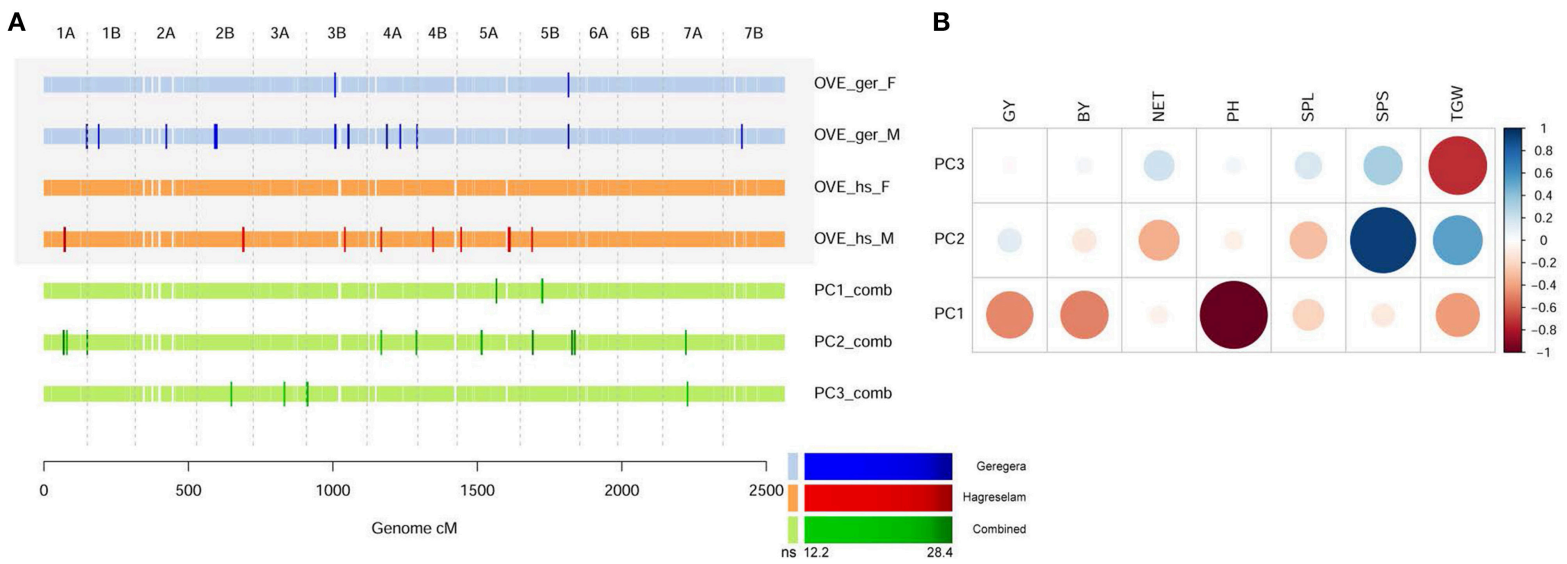
Comparing overall evaluation with principal components of metric traits. **(A)** GWA scan for overall evaluation (OVE) in the two locations, compared to MT-PC 1-3 of combined metric measure of phenotypes. Color code as in Figure 2, the significance of tests as in legend. **(B)** Correlation between the original combined metric values and the derived MT-PC values 1 to 3. Strength and direction of the correlation is represented by circle size and color, respectively. The overall score given by farmers provides a synthetic evaluation of the manifold features of a wheat genotype.

To account for the farmers' holistic evaluation process, we summarized the phenotypic variance of our dataset in a PCA on combined MT values across locations. The first three MT-PCs explained 68.7, 20.1, and 9.4% of the phenotypic variance, respectively, and MTA identified by them showed a varied degree of overlap with those identified by the overall evaluation of farmers (Figure 3A; Table S10). MT-PC1, mostly accounting for PH and yield (Figure 3B), reported two MTAs that were not identified for the FTs in either location. The MTAs identified by MT-PC2, contributed by spike traits and tillering, clearly overlapped with those for the overall evaluation (Figure 3A). Although not always significant, signals on Chr 1A, 3B, and 5B were consistent across overall FT (Figure S6) and MT-PCs (Figure S12). Farmers in the two locations provided different MTAs. Their dissimilarities were clearer when the PCA was performed for each location separately. In this case, the overall measure of the Geregera community identified most of the MTAs detected by local MT-PC1 (Figure S13, Table S11), mostly contributed by PH and yield components (Figure S14). On the other hand, the overall measure of the Hagreselam community, identified several MTAs in common with MT-PC2 on local MTs (Figure S15; Table S12), which was contributed mostly by TGW and spike features (Figure S16).

## Discussion

Farmer scores were independent in Geregera and Hagreselam, as farmers only evaluated their own environment. However, over-locations correlations for combined FTs were high and comparable to those for MTs (Table 1). Farmers were not familiar with the wheat genotypes tested: they were evaluating landraces conserved *ex situ* in unlabeled plots. Therefore, the high correlations detected strongly support the notion that the FTs have a genetic basis. Notably, the overall evaluation provided by farmers scored correlations across environments similar if not higher than that of yield traits in durum wheat (Table 1) and bread wheat alike (Bennett et al., 2012). The high heritability scored by FTs, and particularly by overall, suggests that smallholder farmers' traditional knowledge may indeed be used to guide genetic gain through quantitative methods such as QTL mapping, marker assisted selection, and genomic selection.

Yield and yield components are arguably among the traits most sought after in modern breeding. However, when evaluating varieties in the field, smallholder farmers may prioritize different aspects of the crops (Ceccarelli, 2015). Correlations among FTs and MTs provided the means to break down farmers' appreciation onto quantifiable phenotypes (Table S2). The overall appreciation, the FT more representative of the holistic approach of smallholder farmers in evaluating wheat material, is correlated with a number of metric traits measured in this study, but it cannot be effectively summarized by any of those (Figure S1). The spike morphology FT is also correlated with several MTs, notably thousand grain weight, but similarly to overall it is not collinear with any of those individually (Figure S1). The correlation of MTs with spike and overall may however be used as a proxy of the most valued traits in farmers' opinion among those measured. If one considers overall score as the single most important indicator of farmers' preference, landraces are more frequent than MVs in the top five durum wheat varieties identified in each location (Tab S13). For men in Geregera and Hagreselam, respectively four and three of the top five durum wheat varieties are landraces. Women list four landraces among the top five durum wheat varieties in Geregera, and five landraces out of five in Hagreselam. Women in Geregera and men in Hagreselam list the MV *Bichena* as fourth and second best. Men give the MV *Tossa* the highest overall score among durum wheat varieties in Geregera, and the fifth best in Hagreselem. In all the other cases, landraces are top ranking. Several landraces are jointly identified in the four top-five durum wheat varieties resulting from evaluations given by different genders in different locations. The landrace *DP-228753* is listed among the top five in all cases, but is only 31th for GY and 30th for SPS (Table S3).

The value given to FTs, however, is different across genders and across locations (Figure S1). This results in partially overlapping MTAs identified when operating different data groupings. Earliness is a remarkable example. This FT is inversely related to phenology measures (Figure S1), indicating that farmers consistently prefer early genotypes. Although highly heritable (Table S1) and collinear among genders and locations (Figure 1A), earliness reports two MTAs unique to Hagreselam on Chr 4A and 6A, the latter supported by suggestive peaks in several MTs (Figure S2). The FT scores distribution is different in the two locations (Figure S7), possibly because of the different wheat developmental stage at which the evaluation took place in the two locations. Local pedoclimatic differences may also contribute to contrasting MTA discovered. The length of the wheat life cycle is more important in areas subjected to terminal drought (Kazan and Lyons, 2016), an occurrence more typical of Hagreselam.

Even though tillering capacity was highly correlated with yield traits in both locations (Figure S1, Table S2), this FT was not consistently scored across genders and across locations (Table 1). The corresponding MT, NET, was also scarcely heritable (Table S1), possibly because of marked environmental effects on the expression of the trait. The low *h*^2^ of NET possibly contributed to the fact that we were unable to identify MTAs for farmers preference of tillering capacity. It is also possible that farmers groups provided contrasting evaluations of NET, considering the trait at times positive, at times neutral or even negative.

Spike morphology was perhaps the most important FT in determining the farmers' choice of wheat varieties (Ceccarelli et al., 2000). Spike shape is a good predictor of a number of yield traits in wheat (Gaju et al., 2009), and its evaluation was higly concordant among genders and locations (Figure S3). Although extremely valuable to farmers, yield could not be scored during the open field evaluation: spike was the FT most closely matching it, as shown by its high correlation with yield (Figure S1, Table S2). Whilst seeds per spike (SPS) and spike length (SPL) have clear visual clues on the spike, thousand grain weight (TGW) does not. However, farmers proved to be knowledgeable in identifying several MTAs overlapping TGW putative QTL (Figure 2A). Although some of the spike related traits showed some inflation (Figure S4), a few clear MTAs are consistent in the spike FT and MTs alike. The genotype by environment interaction can alter QTL effects (Boer et al., 2007) and hamper their identification through MTAs even though traits are segregating in single locations, as it is in our case (Table S1). In some cases, a combined analysis over the two locations was necessary to detect MTAs that could not be identified with location-specific MT values. Strikingly, local farmer scores were able to pick up some of these MTAs (Figure 2A). This is the case, for example, of the MTA on Chr 1A for Geregera spike morphology FT and for combined measures of TGW. Farmers were able to detect similar MTAs on Chr 2A and Chr 4A for TGW and SPS, respectively (Figure 2A). Farmers' traditional knowledge, elicited through FTs, is the result of their past field experiences: unlike metric values, it builds on the time dimension, considering altogether the field conditions over time under which genotypes were grown.

Although tightly linked to overall evaluation (Figure S1), spike morphology is not the sole trait contributing to it, thus the two traits provide only partially overlapping MTAs (Figure 2). The measure of grain yield combined over locations identify an MTA on Chr 5B with clear overlap to overall FT signals in both locations. This MTA co-maps with an MTA for SPS and spike FT, and confirms the importance of production in determining the desirability of a genotype. The overall score summarizes the farmer's view of the value of the variety and potentially his or her willingness to invest resources in growing it. When providing an overall evaluation of the plot, farmers were simultaneously scoring and weighting a multitude of traits, probably exceeding those measured in MTs. This possibly contributes to the poor overlap between MTAs deriving from the overall evaluation and those deriving from the PCA conducted on MTs (Figure 3). When compared within locations, however, MTAs deriving from MT-PCs were clearly overlapping with the overall FT. This is especially true in Geregera, where all but four MTAs identified by the farmers were also detected by the MT-PC1 deriving from MTs (Figure S13). This suggests that farmers' overall evaluation, that is independent from the metric measures collected, can indeed provide a synthetic evaluation of the many traits that make up a wheat ideotype according to farmers. The combination of traits inducing farmers' appreciation is determined by their traditional knowledge, and it is hardly ascribable to categorical metric phenotypes: smallholder farmers' overall evaluation depends at all times on their environmental and cultural background, and is likely influenced by metric as well as qualitative traits beyond those measured in this study.

Although both men and women farmers were chosen because they were wheat growers, we found that different genders may evaluate FTs differently. The women groups provided more skewed and varied evaluations for all FT, especially in Hagreselam (Figure S7), possibly lowering their MTA detection power (Table S1) and resulting in less putative QTL identified as compared with male farmers. It is likely that women and men differently evaluate wheat traits, especially in regards to quality vs. productivity (Defoer et al., 1997; Assefa et al., 2014). This may contribute to the discrepancy we observed in FTs evaluated between genders. In fact, even though farmers cannot evaluate quality traits while in the field, they may select for spikes and plants resembling those that in their past experience provided good flour for specific food and drink preparations. Further studies extending the spectrum of measured FTs and MTs beyond field traits are needed to explore genetic basis of farmers' perception of quality traits and its relation to farmers' choice of materials.

In the present study each farming community evaluated only one field for one year, hence we could not perform a detailed study over genotype by environment interactions. However, the high correlation among traits across locations (Table S2) and the overlap of putative QTL deriving from MTs and FTs collected across locations (Table S8) support the relevance of the MTAs we identified. As the number of MTA per putative QTL increases, so does the overlap of putative QTL identified by MTs and FTs (Figure S11). This is likely contributed by local regions of higher LD extent increasing the span of the significance interval identified by MTAs. Interestingly, most of putative QTL identified by MTs are also detected by FTs. This finding, joined with the high phenotyping variance explained by some of the FT MTAs (Table S9), further supports the use of smallholder farmers evaluations as mean to identify genomic loci relevant for marker assisted breeding.

The detailed discussion of the putative QTL we identified is beyond the aims of this study. Several reasons prevent us from doing so. Although a gold standard for wheat genotyping, the genomic coverage provided by the 90,000 markers employed (Wang S. et al., 2014) is still sparse on the vast wheat genome. At the same time, the uniqueness of Ethiopian wheat (Mengistu et al., 2016) puts the panel employed aside the literature already existing on the topic. After the release of draft genome sequences (Brenchley et al., 2012; Mayer et al., 2014), more advanced genomic tools are being developed for durum and bread wheat alike, and will allow the finer dissection of putative QTL and guide the identification of candidate regions. In particular, the study of genotype by environment interaction of FT evaluations will allow to better characterize the relevance of the putative QTL we identified in a breeding perspective. It should be noted that smallholder farmers typically grow mixtures of genotypes to better cope with unpredictable adverse growing conditions. Indeed landraces are typically heterogeneous. Further studies exploring the variability of germplasm maintained *in situ* are needed to better understand the relationship of farmers' choice and genetic diversity in their fields, so to better exploit the functional diversity found within traditional materials.

Our survey highlights that smallholder farmers' evaluations are consistent and target measurable quantities. Because of this, farmers are capable of identifying MTAs for traits of their interest through FTs, in some cases in a gender and locality-dependent way. In many other cases, trans-location farmers' MTAs are independently targeted, and elude our classical phenotyping. Our results show that it is feasible to involve farming communities to directly evaluate broad collections of genotypes using a selected set of summary traits previously agreed. In fact, during the 2016 growing season, with an effort requiring 2 weeks of field work, smallholder farmers from a third community in the Amhara region of Ethiopia evaluated 1,200 recombinant inbred lines we produced from the diversity panel here employed. By scaling up the approaches here introduced, we aim to speed up the genetic gain in breeding targeting smallholder farming systems. We advocate the employment of our method in different genotypes and crops, agro-ecologies, and smallholder farming communities to connect participatory variety selection to modern plant breeding, ultimately allowing the production of MVs more closely addressing smallholder farmers' needs. The current and upcoming genomic tools enable breeding to take advantage of the unique knowledge that smallholder farmers have gathered in thousands of years of cropping of available genetic resources: traditional knowledge coming from the past could propel the breeding of the future.

## Author contributions

MP and CF originally conceived the experiment. CF, YK, and DM identified and enrolled the farming communities. YK, DM, and CM conducted focus group discussion and supervised field evaluations. MP and MD supervised data analyses. YK, CM, and EF analyzed metric traits and farmer traits. MD conducted genotypic and statistical data analysis, produced figures, and drafted the manuscript. All of the authors critically revised and approved the final manuscript.

### Conflict of interest statement

The authors declare that the research was conducted in the absence of any commercial or financial relationships that could be construed as a potential conflict of interest.
