# Supplementary material for: Genome Wide Association Study to Identify the Genetic Base of Smallholder Farmer Preferences of Durum Wheat Traits
Source: Front Plant Sci. 2017 Jul 17;8:1230. doi: 10.3389/fpls.2017.01230 (PMC5511852; doi:10.3389/fpls.2017.01230)
Supplement: Supplementary file 15 [file Presentation1.zip › Supplemental_figures/S4_Fig.pdf]

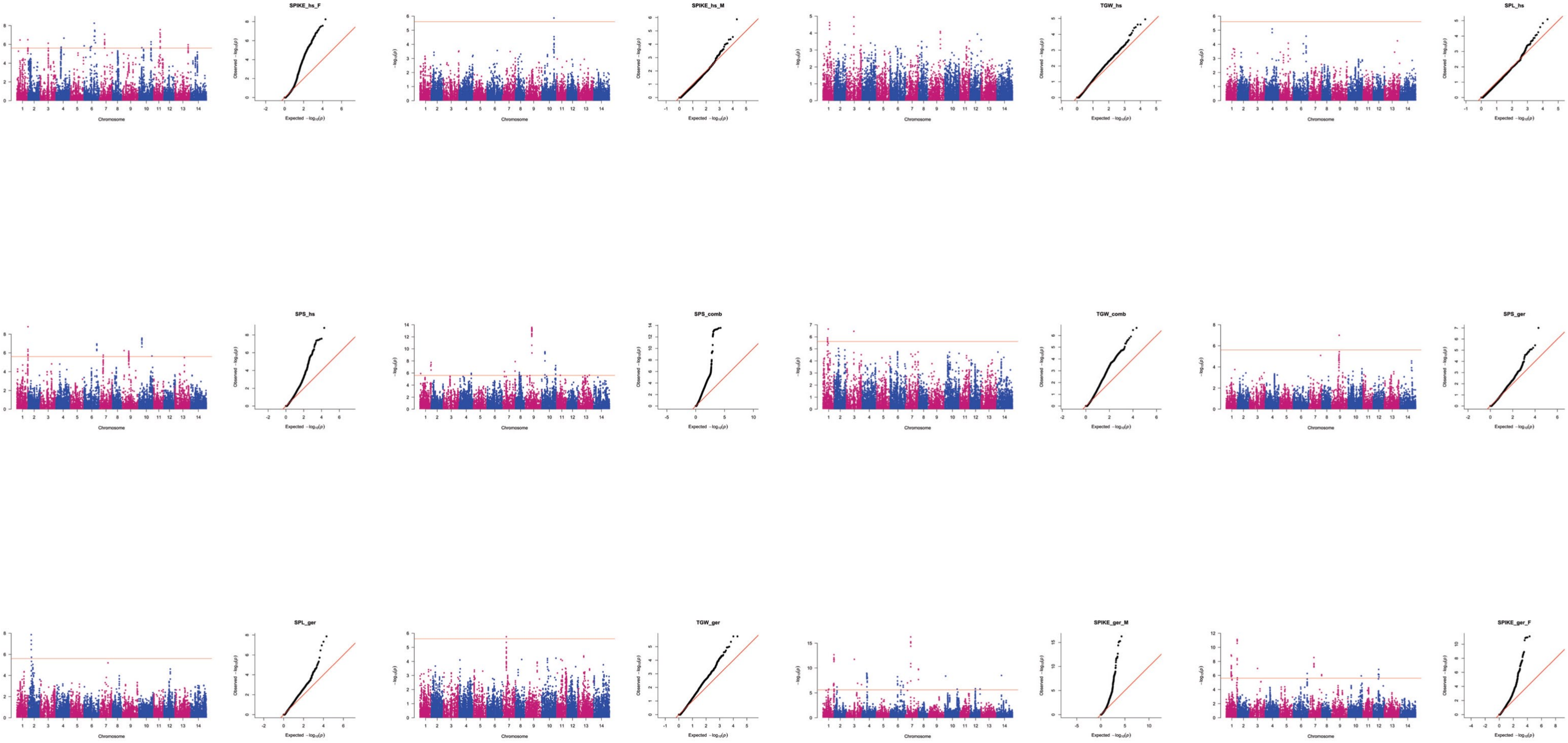

Supplementary Figure 4. GWA results for spike FT and related MTs. A Manhattan plot and a quantile-quantile plot are given for each GWA scan. Traits names are listed top right. In the Manhattan plot, chromosomes are ordered 1 to 14 (from 1A to 7B). Points represent markers, depicted in alternating colors according to chromosomes. On the x-axis, the genomic position of markers. On the y-axis, the negative logarithm of the significance of the association test. The red threshold represents the Bonferroni correction for a nomil test p value of 0.05. Quantile-quantile plots report the observed (y-axis) versus the expected (x-axis) p value distribution for individual tests (black dots). The red line represents the null model.
