# Supplementary figures and images for: Genome Wide Association Study to Identify the Genetic Base of Smallholder Farmer Preferences of Durum Wheat Traits
Source: Front Plant Sci. 2017 Jul 17;8:1230. doi: 10.3389/fpls.2017.01230 (PMC5511852; doi:10.3389/fpls.2017.01230)

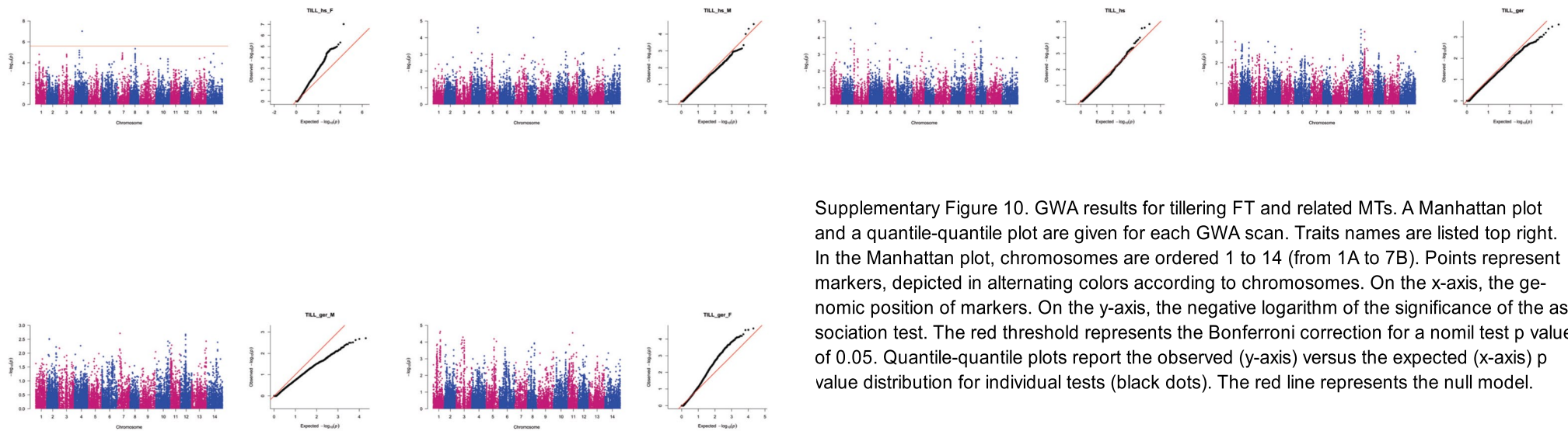

Supplement: Supplementary file 15 [file Presentation1.zip › Supplemental_figures/S10_Fig.pdf]

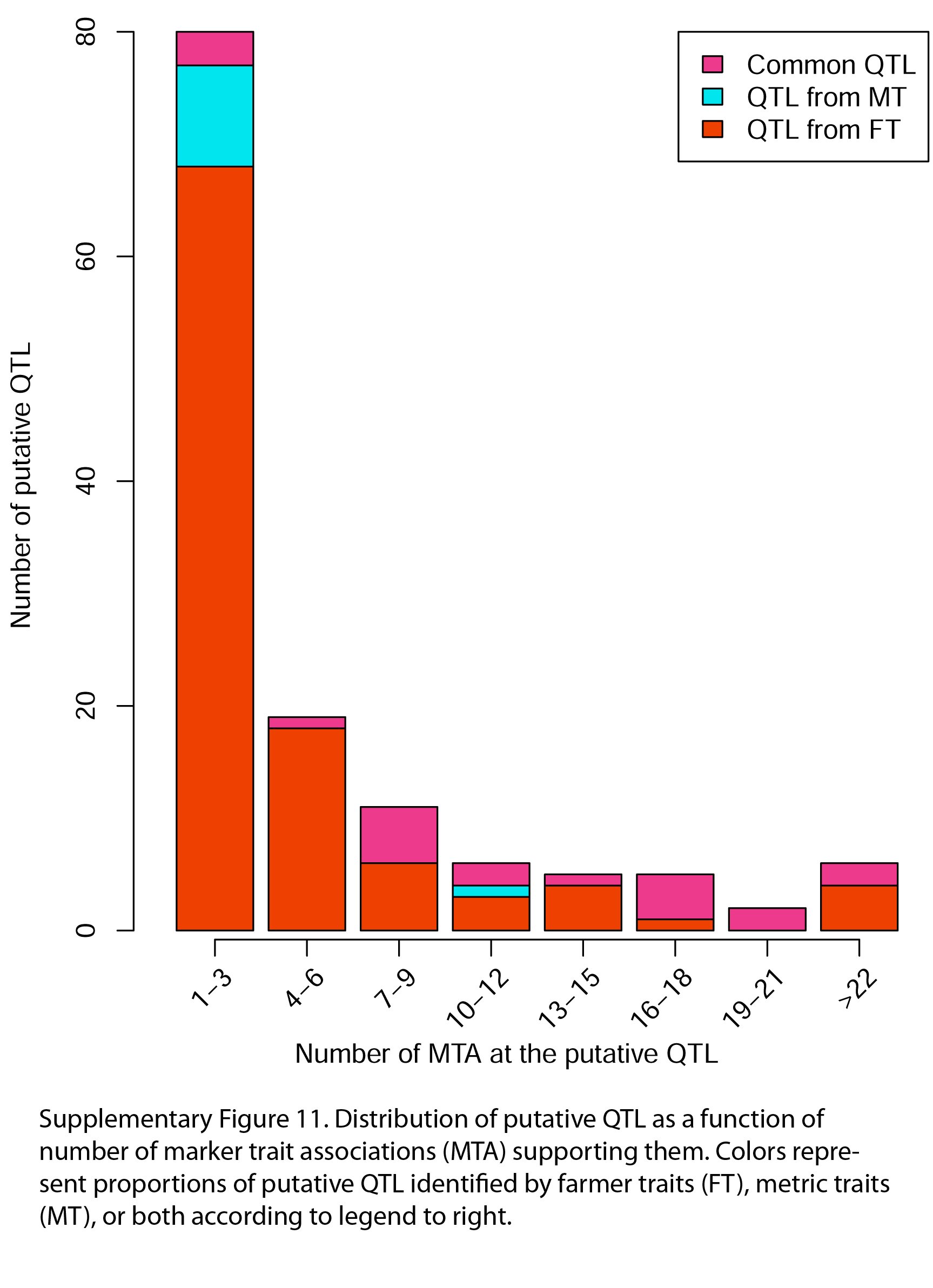

Supplement: Supplementary file 15 [file Presentation1.zip › Supplemental_figures/S11_Fig.tif]

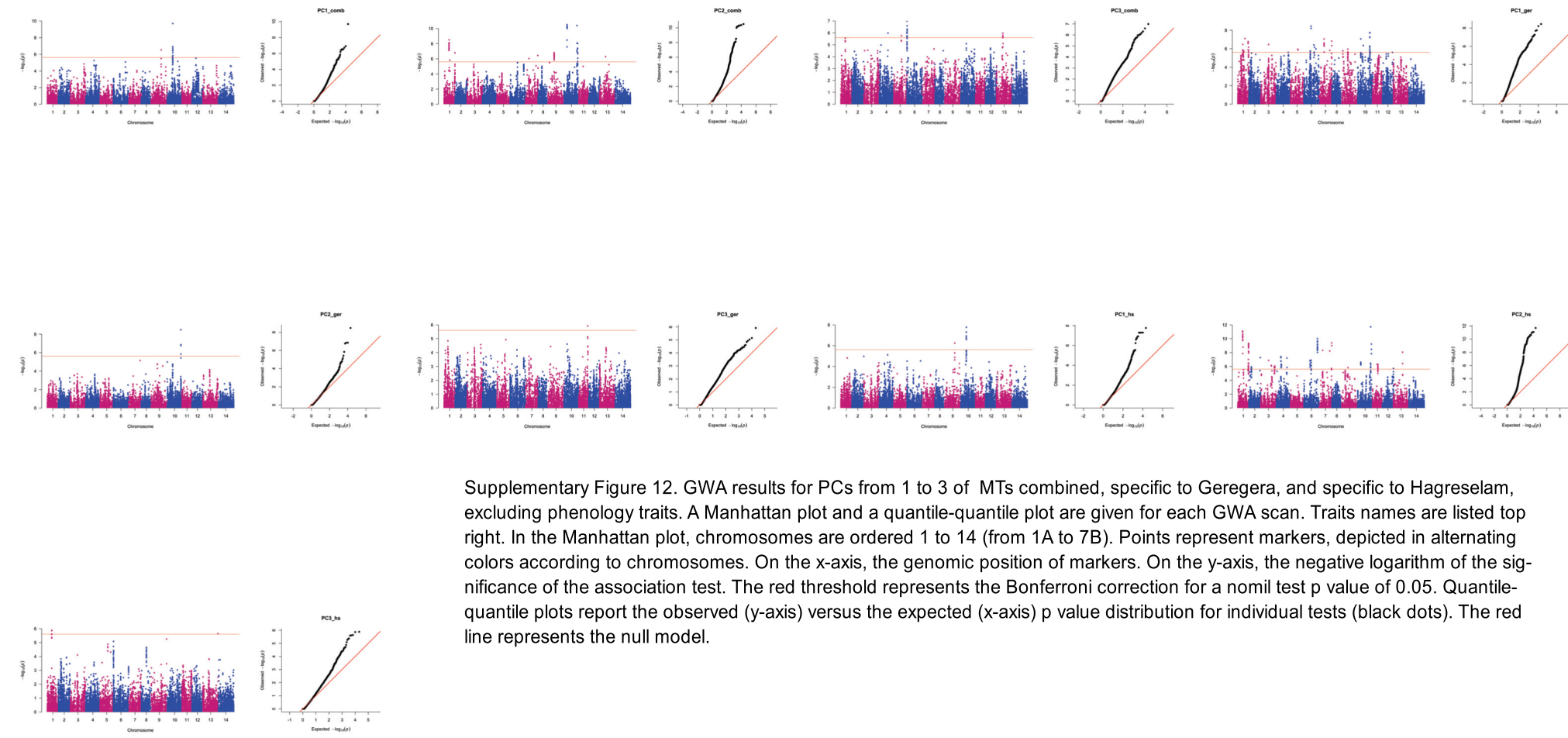

Supplement: Supplementary file 15 [file Presentation1.zip › Supplemental_figures/S12_Fig.pdf]

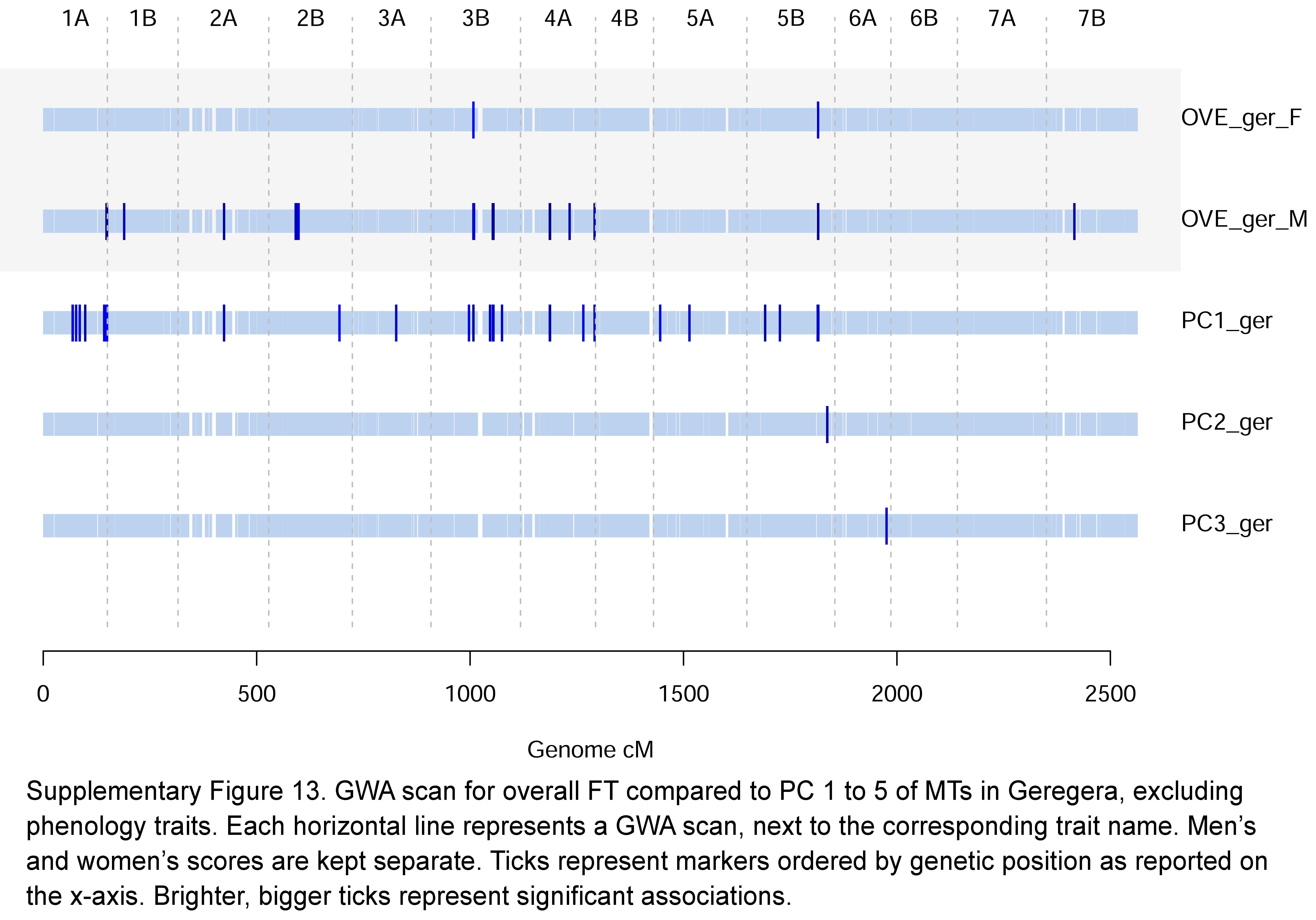

Supplement: Supplementary file 15 [file Presentation1.zip › Supplemental_figures/S13_Fig.tif]

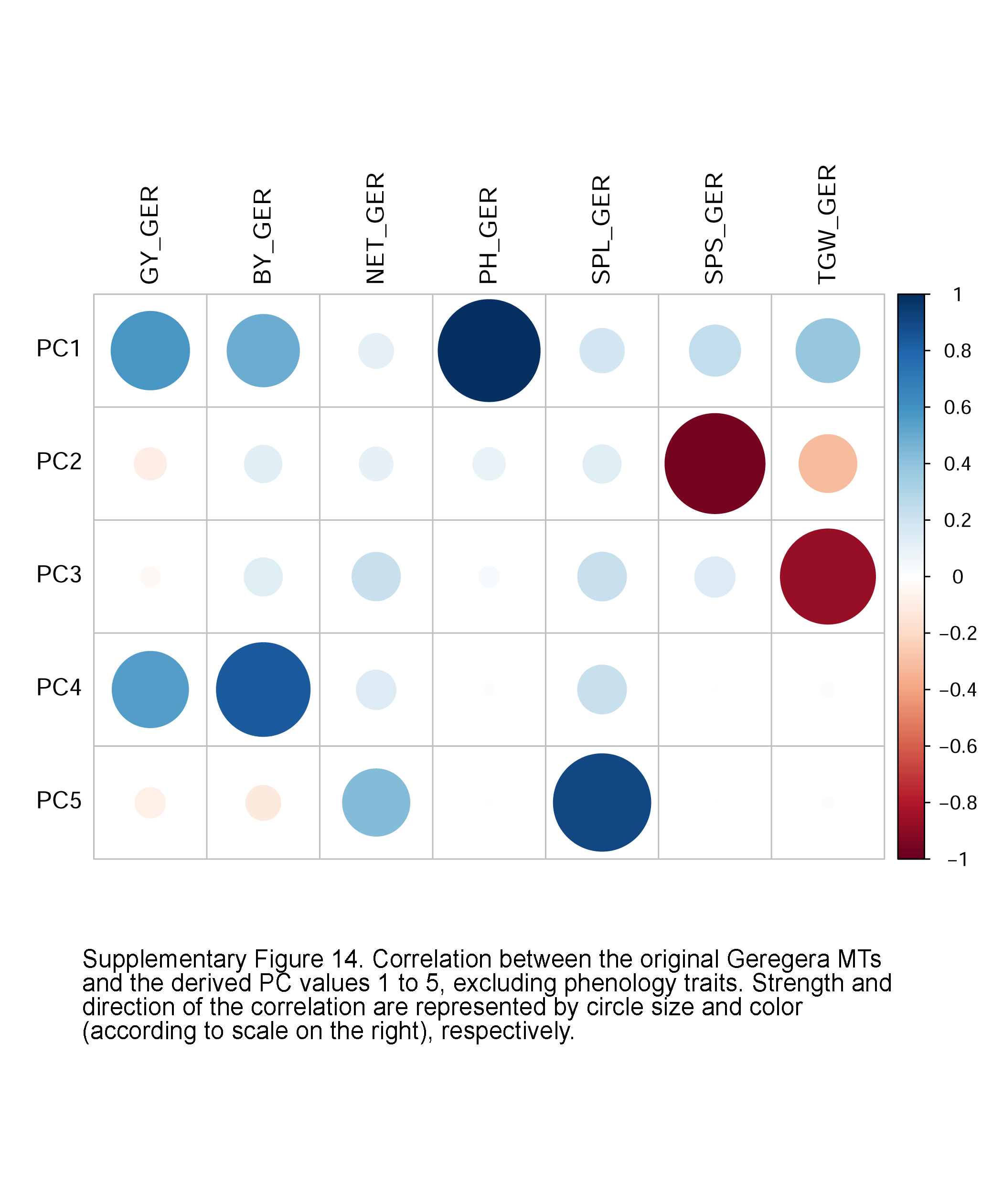

Supplement: Supplementary file 15 [file Presentation1.zip › Supplemental_figures/S14_Fig.tif]

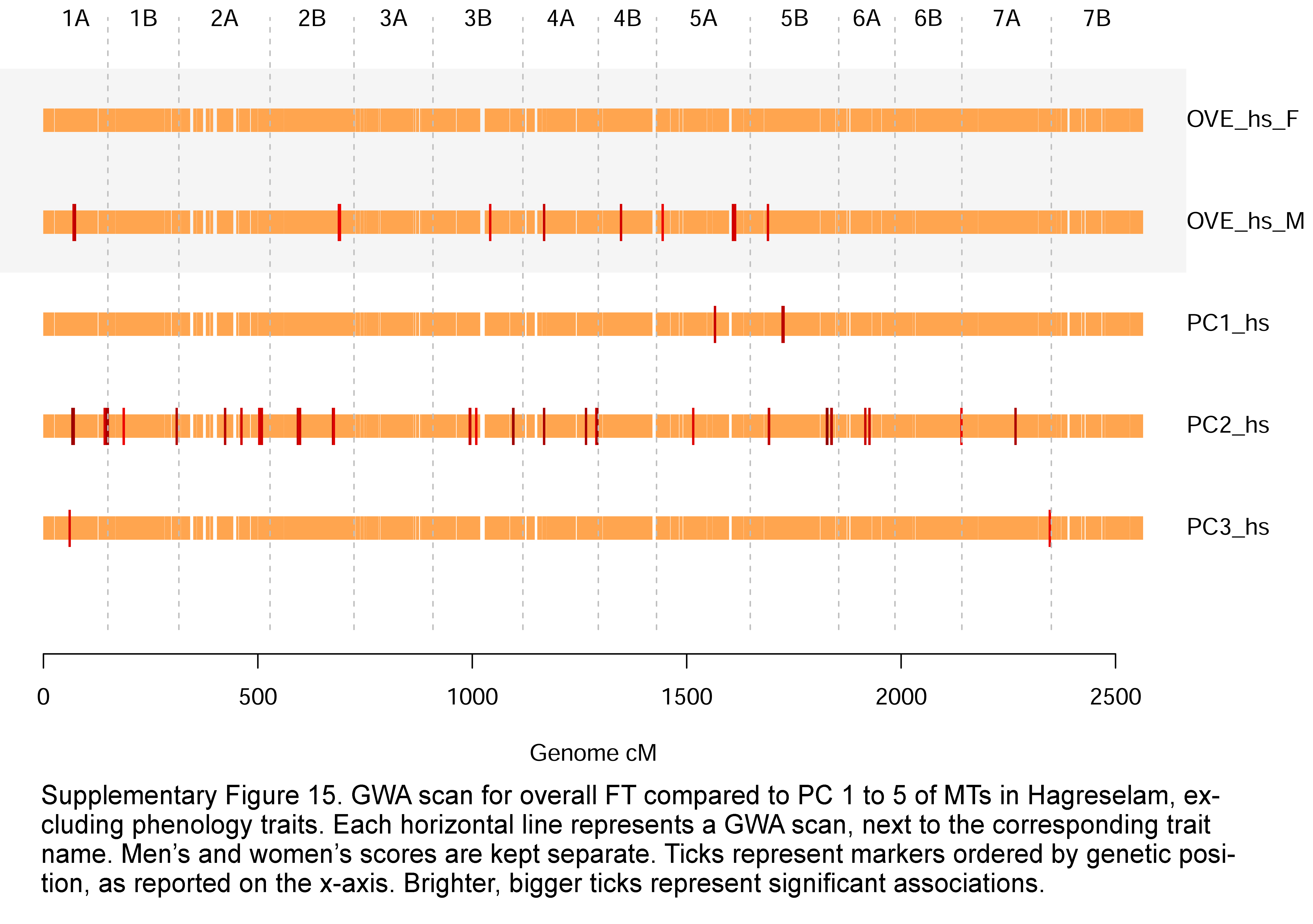

Supplement: Supplementary file 15 [file Presentation1.zip › Supplemental_figures/S15_Fig.tif]

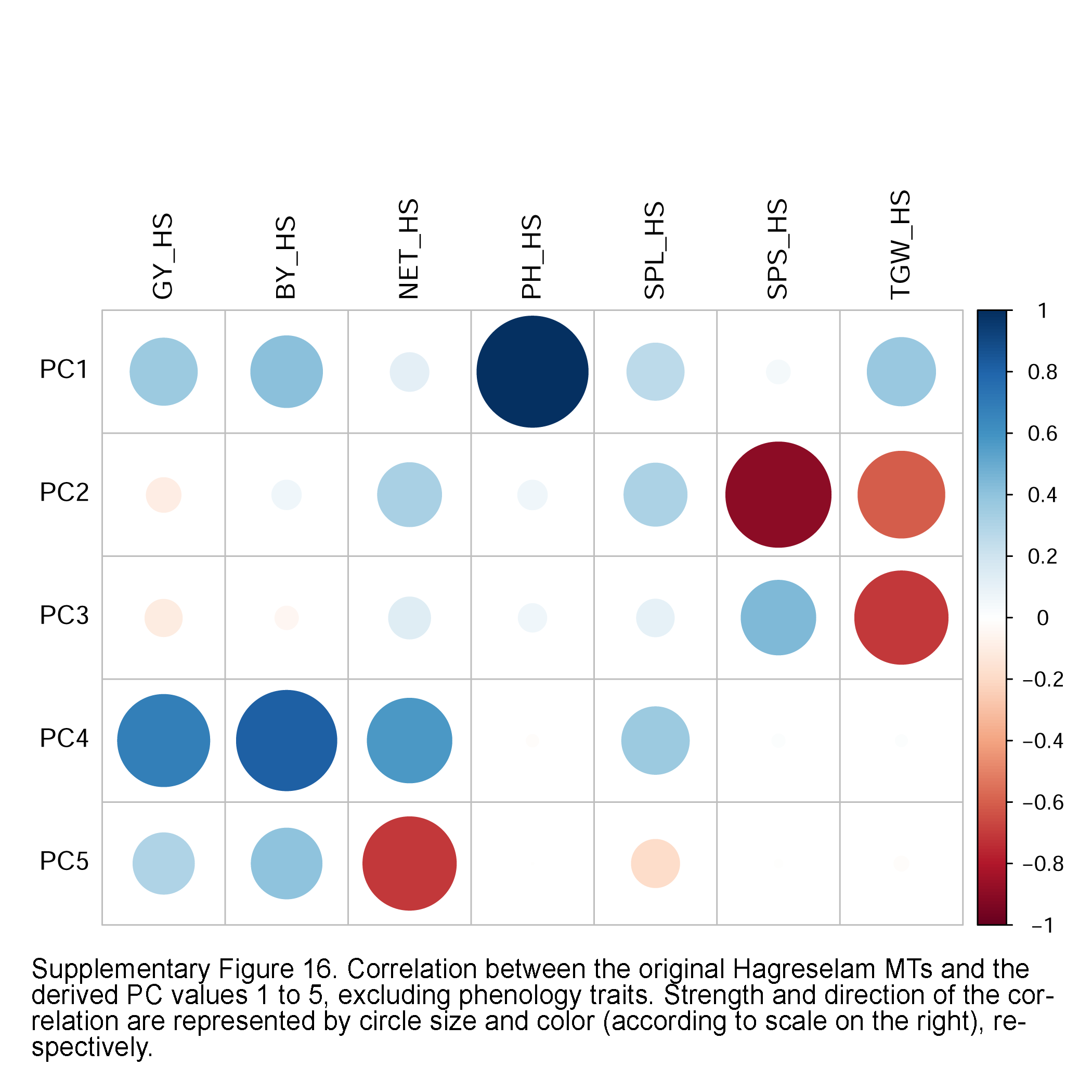

Supplement: Supplementary file 15 [file Presentation1.zip › Supplemental_figures/S16_Fig.tif]

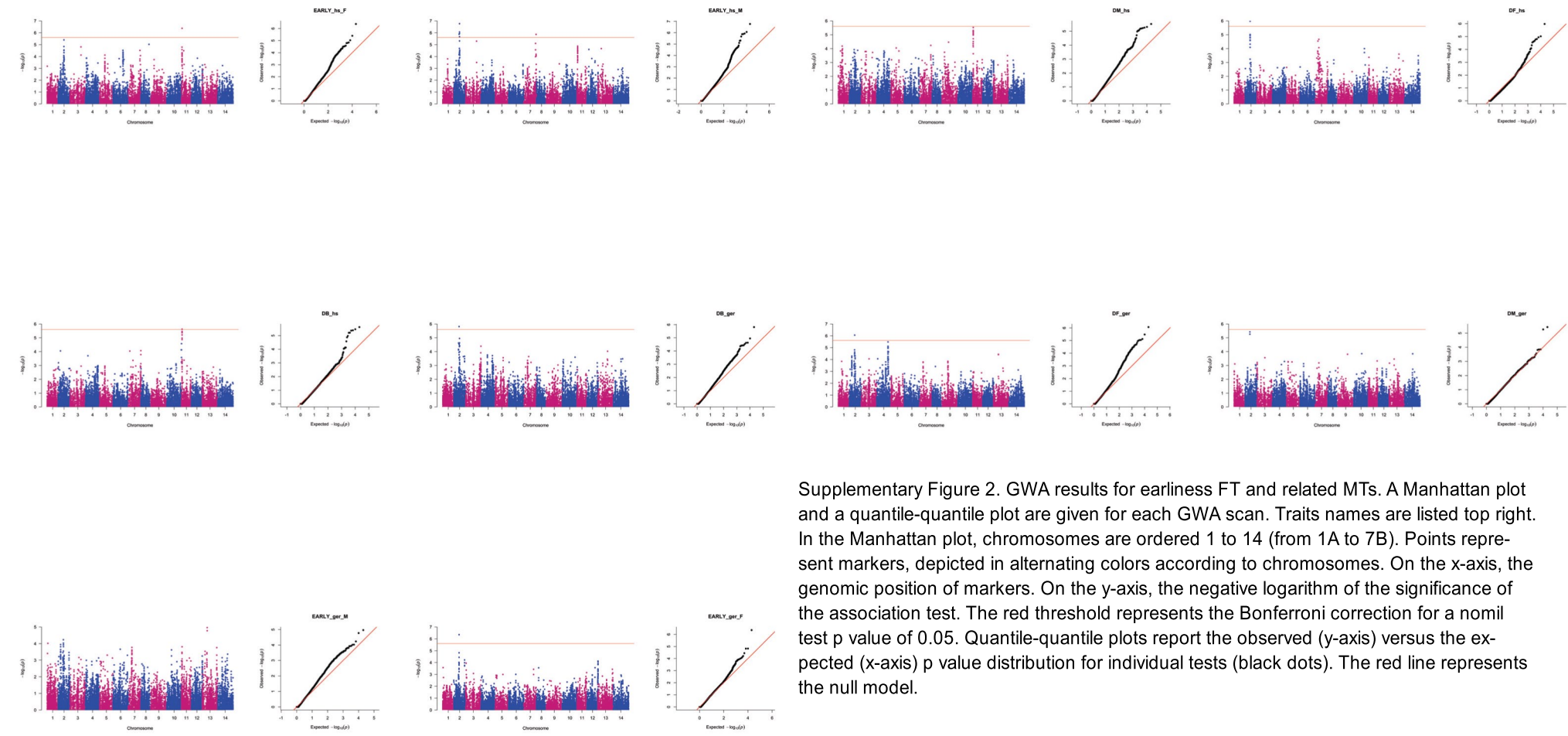

Supplement: Supplementary file 15 [file Presentation1.zip › Supplemental_figures/S2_Fig.pdf]

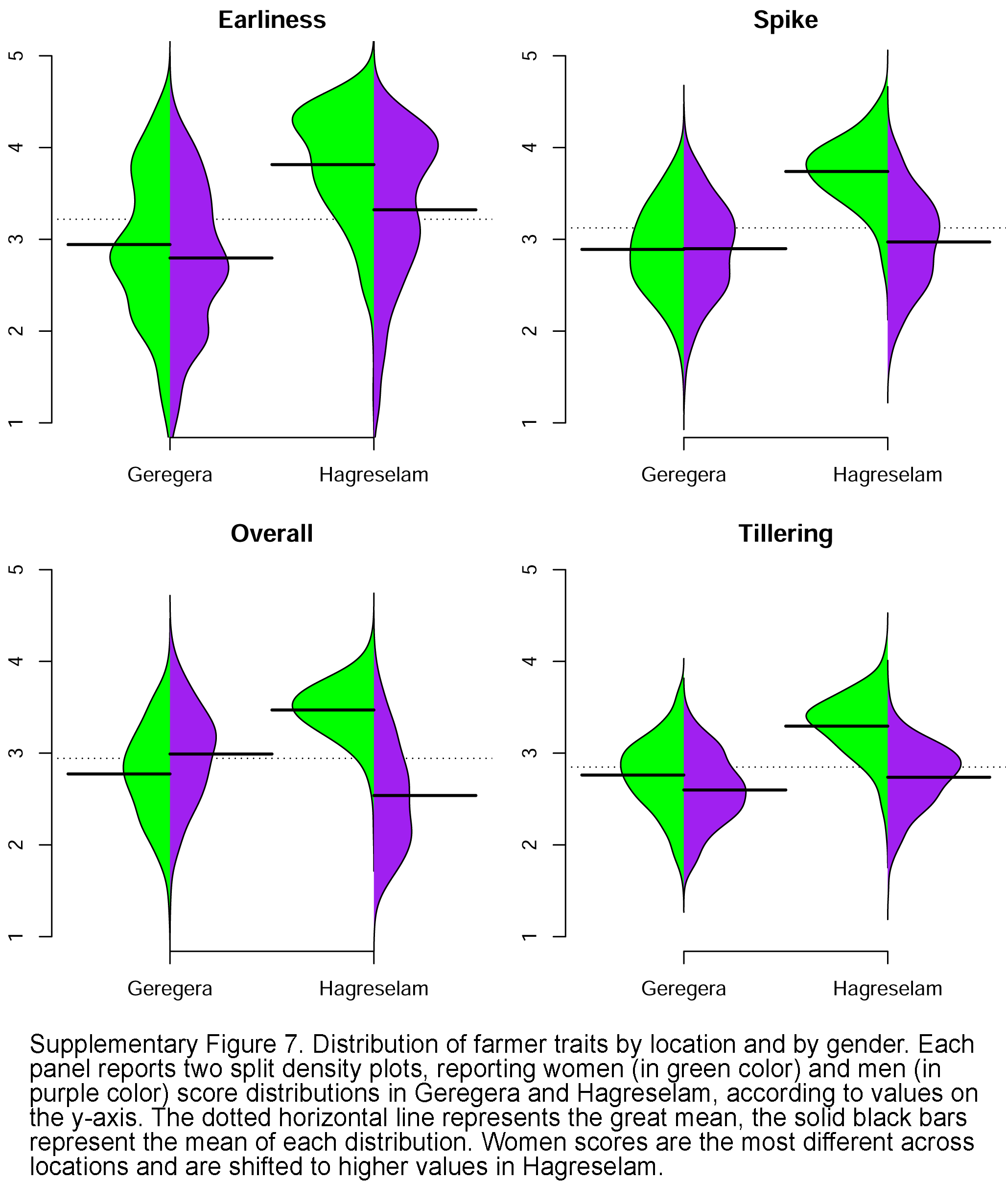

Supplement: Supplementary file 15 [file Presentation1.zip › Supplemental_figures/S7_Fig.tif]

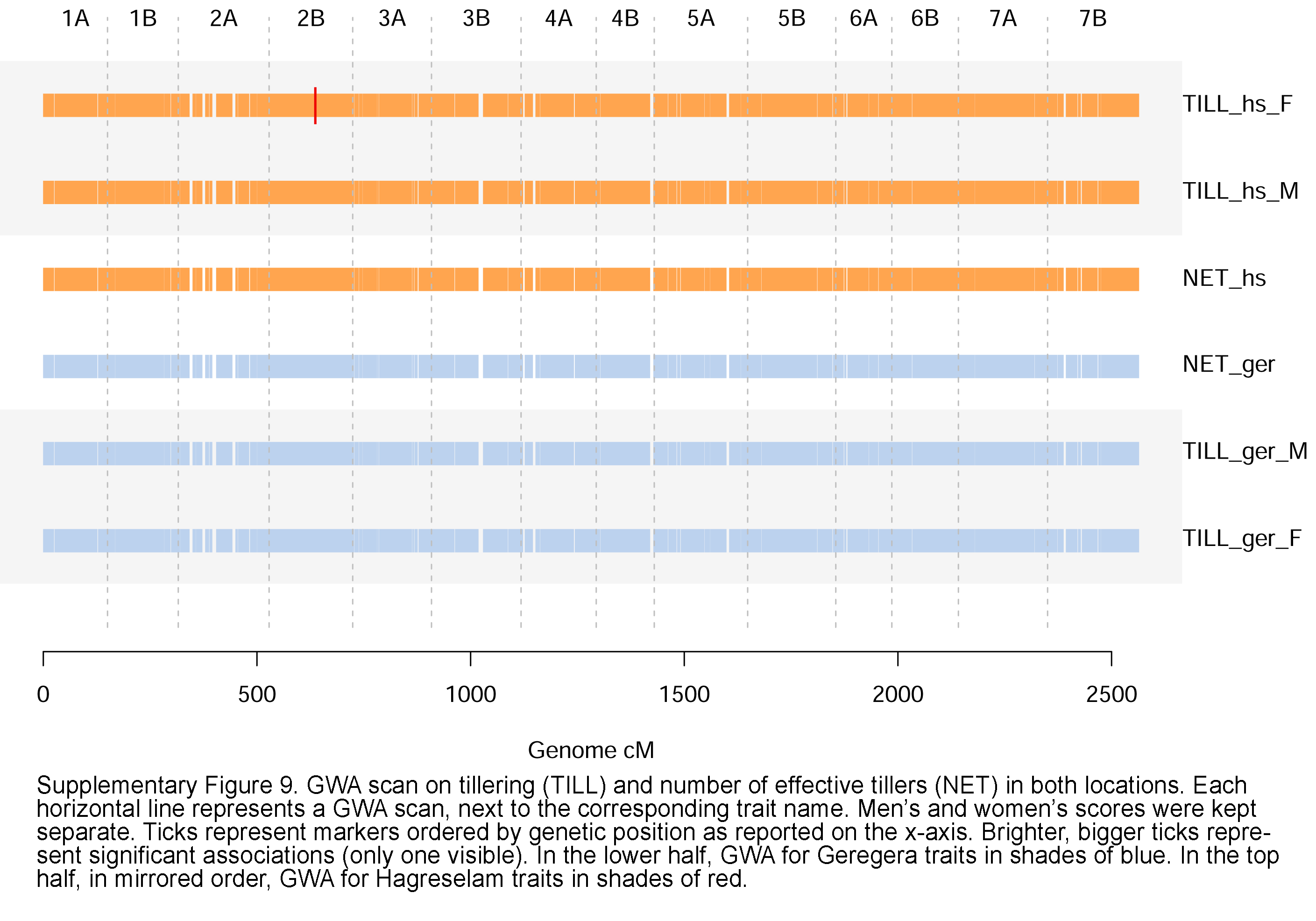

Supplement: Supplementary file 15 [file Presentation1.zip › Supplemental_figures/S9_Fig.tif]
